# Disparities in Healthcare Utilization Among Vulnerable Populations During the COVID-19 Pandemic in Brazil: An Intersectional Analysis

**DOI:** 10.3390/ijerph22060831

**Published:** 2025-05-25

**Authors:** Letícia Perticarrara Ferezin, Rander Junior Rosa, Heriederson Sávio Dias Moura, Mônica Chiodi Toscano de Campos, Felipe Mendes Delpino, Murilo César do Nascimento, Juliana Soares Tenório de Araújo, Ione Carvalho Pinto, Ricardo Alexandre Arcêncio

**Affiliations:** 1 Department of Maternal and Child Nursing and Public Health, School of Nursing, University of São Paulo at Ribeirão Preto, São Paulo 05508-220, Brazil; rander1junior@usp.br (R.J.R.); heriederson@usp.br (H.S.D.M.); julianastenorio17@gmail.com (J.S.T.d.A.); ionecarv@eerp.usp.br (I.C.P.); ricardo@eerp.usp.br (R.A.A.); 2 Department of Nursing, School of Health Sciences, University of Brasília, Brasília 70910-900, Brazil; monicachiodi@unb.br; 3 Graduate Program in Dentistry, Federal University of Pelotas, Pelotas 96010-610, Brazil; fmdsocial@outlook.com; 4 Graduate Program in Nursing, Nursing School, Federal University of Alfenas, Alfenas 37130-001, Brazil; murilo.nascimento@unifal-mg.edu.br

**Keywords:** COVID-19, social vulnerability, intersectional framework, public health services

## Abstract

Background: Brazil’s Unified Health System (Sistema Único de Saúde—SUS) has played a crucial role in reducing health disparities by providing universal and free healthcare to a diverse population. However, the COVID-19 pandemic exposed significant barriers to healthcare access among vulnerable groups, particularly due to the intersection of multiple vulnerabilities. This study aimed to examine how intersectionality—specifically sex/gender, race/ethnicity, and education level—has influenced inequalities in healthcare service utilization among vulnerable populations during the COVID-19 pandemic in Brazil. Methods: This cross-sectional study is part of the “COVID-19 Social Thermometer in Brazil” project, conducted between May 2022 and October 2023 in Brazil’s state capitals and the Federal District, focusing on populations considered socially vulnerable during the pandemic. Participants were selected using sequential sampling and completed a structured questionnaire. Statistical analyses—performed using Excel, RStudio (version 4.3.2), and ArcGIS—included sociodemographic profiling, the construction of the Jeopardy Index (a measure of social vulnerability), and binary logistic regression to explore associations between Jeopardy Index and healthcare service utilization. Results: 3406 participants, the majority were men (60%), aged 30 to 59 years (65.1%), and identified as Black or Brown (72.2%). Most participants were concentrated in the Northeast (26.6%) and North (22.3%) macroregions. A high reliance on public healthcare services (SUS) was observed, particularly in the Southeast (96%). According to the Jeopardy Index, the most socially vulnerable groups—such as women, transgender individuals, Black people, and those with no formal education—were significantly more likely to rely on SUS (OR = 3.14; 95% CI: 1.34–7.35) and less likely to use private healthcare (OR = 0.07; 95% CI: 0.02–0.20), reflecting a 214% higher likelihood of SUS use and a 93% lower likelihood of private service utilization compared to the most privileged group. Conclusions: Our findings reveal that individuals experiencing intersecting social vulnerabilities face marked inequalities in healthcare access. Without SUS, these populations would likely have been excluded from essential care. Strengthening SUS and implementing inclusive public policies are critical to reducing disparities and ensuring equitable healthcare access for historically marginalized groups.

## 1. Introduction

The COVID-19 pandemic exposed the deep social and economic inequalities present in societies worldwide, highlighting the increased vulnerability of certain population groups to the impacts of health crises [1]. In Brazil, where the estimated population in 2021 was 213 million, according to the Brazilian Institute of Geography and Statistics, around 60 million people lived in poverty, representing approximately 37% of the population [2].

While the virus affected all social strata, its consequences were not equally distributed [3]. Historically marginalized groups—such as Black and Indigenous people, LGBTQIA+ individuals, women, and residents of favelas and peripheral areas—have faced additional barriers to accessing and utilizing healthcare services, which were further exacerbated during the pandemic [3,4].

Inequality has been a persistent historical and social issue in Brazil since colonization, with recent data highlighting the significant and enduring presence of structural racial disparities across Brazilian society. In 2019, the poverty rate among the Black population was 32.9%, nearly double that of the White population (15.4%). Black women earned, on average, only 44% of the income earned by White men, reflecting the intersection of racial and gender inequalities. In education, only 5.6% of Indigenous people and 8.8% of Black individuals had completed higher education, compared to 22% of the White population [5].

In terms of healthcare, reliance on the Unified Health System (SUS) is inversely proportional to income: 86% of the poorest 25% of the population depend exclusively on SUS, compared to only 25% of the wealthiest 10% [6]. During the pandemic, these disparities worsened, with the COVID-19 mortality rate being 1.5 times higher among Black individuals than among White individuals [7].

There is an interaction among these vulnerabilities that further complicates the COVID-19 situation. The author Kimberlé Crenshaw describes this in her theory of intersectionality: when a person belongs to multiple marginalized groups—such as those defined by race, gender, class, or disability—these identities do not operate independently but intersect to create unique forms of discrimination and exclusion. This intersectionality can reduce access to healthcare and increase overall vulnerability [8].

In many contexts, overlapping forms of inequality have worsened health outcomes, leading to situations where existing challenges were significantly amplified [5,6]. Although Brazil has adopted a universal health system, known as SUS, and care is theoretically provided free of charge to all, this care has unfortunately not been accessible to vulnerable populations, who suffered most acutely during the pandemic [9,10].

According to the literature, vulnerable populations are groups of individuals who, due to social, economic, or historical disadvantages, are more likely to experience exclusion and lack protection [11,12]. The World Health Organization (WHO) explains that vulnerability often entails facing obstacles in accessing essential services, being more exposed to environmental or occupational risks, and having fewer resources to cope with emergencies [13].

In Brazil, the Ministry of Health considers individuals vulnerable if they struggle to access healthcare due to factors such as poverty, geographic location, cultural differences, or institutional barriers. This includes people who are homeless, individuals with disabilities, Indigenous peoples, Quilombola communities, and other groups that have historically faced discrimination or marginalization [14,15]. In addition to socioeconomic factors, geographic location—such as living in rural or remote areas—creates further physical barriers to accessing healthcare services, thereby increasing vulnerability [16].

Brazil’s healthcare system comprises two main components: the public SUS, which serves approximately 75.5% of the population—primarily those with lower incomes—and the private sector, which caters to wealthier individuals who can afford health insurance or pay out of pocket for services. This structure underscores significant socioeconomic disparities in access to healthcare and exclusion [17,18]. Socioeconomic stratification also risks weakening the political influence and collective advocacy needed to drive improvements in the public system, as the absence of higher-income stakeholders can undermine both the system’s accountability and its capacity for reform [19].

The SUS faces severe underfunding, with public spending accounting for only 44% of total health expenditures, while private spending makes up 56%. This dual structure results in high-income patients relying on private services for routine care but turning to the SUS for complex treatments, contributing to system overload and inequitable resource distribution [20,21]. Regional disparities further compound these challenges, as chronic underinvestment in healthcare infrastructure in the North and Northeast—particularly in rural areas—continues to limit access to primary healthcare [22].

Analyzing healthcare access through the lens of health service utilization during the COVID-19 pandemic—from an intersectional perspective focused on vulnerable populations—is essential for understanding inequalities and strengthening public policies that promote equity in care.

The concept of healthcare access involves ensuring that individuals and populations can obtain appropriate health services when needed, without encountering disproportionate barriers. According to the WHO, healthcare access is defined across four key dimensions: availability, referring to the presence of sufficient services and resources to meet the population’s needs; accessibility, encompassing physical, financial, and geographical factors that facilitate or hinder the use of these services; acceptability, which assesses whether services are socially and culturally appropriate, respecting users’ characteristics and values; and quality, ensuring that services are effective, safe, and evidence-based. These dimensions are fundamental to achieving equity in healthcare access and to realizing the right to health [23].

However, ensuring access to healthcare services does not necessarily translate into their effective utilization. The use of these services is shaped by a complex interplay of individual and collective factors, including perceived need, awareness of available services, sociocultural and organizational barriers, and prior experiences with the healthcare system [24]. This phenomenon may be influenced by the intersectionality of vulnerabilities, highlighting the need for targeted approaches and further investigation into this issue.

A review of the scientific literature revealed no studies conducted in Brazil that specifically examined the role of the SUS in mitigating barriers and strengthening the utilization of healthcare services through an intersectional lens. The use of an intersectional approach in studies on this topic remains limited. This perspective—which considers the interaction of multiple social factors—is essential for understanding inequalities among vulnerable populations and for reinforcing the SUS. Therefore, this study aimed to investigate how intersectionality—considering sex/gender, race/color, and education level—has influenced inequalities in the utilization of healthcare services by vulnerable populations during the COVID-19 pandemic in Brazil.

## 2. Materials and Methods

### 2.1. Study Design

This is a cross-sectional study [25,26], based on field interviews conducted between May 2022 and October 2023 in the capitals of Brazil’s 26 federal units (UFs) and the Federal District. The study is part of a larger research project titled “COVID-19 Social Thermometer in Brazil.”

The COVID-19 pandemic period, according to the WHO, began on 11 March 2020, when the global spread of SARS-CoV-2 was officially declared a pandemic. This period marks the most critical phase of impact on healthcare systems worldwide. Although the WHO declared the end of the Public Health Emergency of International Concern on 5 May 2023, the effects of the pandemic continue to persist, highlighting the importance of sustaining efforts to protect the most vulnerable populations [27].

During the pandemic, the COVID-19 mortality rate in Brazil was 1.5 times higher among Black individuals compared to White individuals [28]. Previous studies have shown that this disparity reflects the historical and structural inequalities affecting the Black population in Brazil, including social exclusion, unequal access to quality healthcare, and precarious living conditions—factors that increase their vulnerability to severe illnesses [29].

In Brazil, the process of easing COVID-19-related restrictions began gradually in 2021, driven by the progress of the vaccination campaign and a decline in severe cases and deaths [30]. Between mid-2021 and early 2022, schools, commercial establishments, and cultural and sporting events reopened, albeit with capacity limits and continued implementation of safety protocols, such as mandatory mask use [31]. Given this transitional context, field interviews began only in May 2022.

Brazil, located in South America, is the fifth-largest country in the world, covering 8,510,417.771 km². It is the largest country in South America and the third-largest in the Americas, accounting for about 50% of the continent’s total area. The country is divided into five macroregions:

− North (Acre, Amapá, Amazonas, Pará, Rondônia, Roraima, and Tocantins),
− Northeast (Alagoas, Bahia, Ceará, Maranhão, Paraíba, Pernambuco, Piauí, Rio Grande do Norte, and Sergipe),
− Midwest (Goiás, Mato Grosso, and Mato Grosso do Sul),
− Southeast (São Paulo, Minas Gerais, Rio de Janeiro, and Espírito Santo), and
− South (Paraná, Santa Catarina, and Rio Grande do Sul).

This regional division is intended to optimize administrative organization and public policy planning, while also respecting the unique characteristics of each region [32].

### 2.2. Study Population

The study population consisted of individuals aged 18 or older who belonged to one of four vulnerable population groups in Brazil, as follows:

(a) homeless individuals—native or naturalized Brazilians who had lived on public streets, in shelters, or in similar locations designated for this population for at least six months during the COVID-19 pandemic;
(b) migrants and refugees—individuals from other countries who had resided in Brazil for at least six months during the pandemic and had basic proficiency in interpreting or understanding the Portuguese language;
(c) residents of slums—native or naturalized Brazilians living in urban areas characterized by inadequate housing and poor infrastructure (such as favelas, informal settlements, or urban occupations) during the pandemic;
(d) residents of camps, settlements, or occupations—native or naturalized Brazilians who had lived in these areas for at least six months during the pandemic.

The exclusion criterion was the omission of key responses, specifically the failure to complete the questionnaire or to indicate the specific vulnerable population group to which the participant belonged.

### 2.3. Sampling

Given the nature of the studied populations—particularly their social and institutional invisibility—participant selection was carried out using sequential sampling [33]. In this approach, individuals were selected based on their location and availability to participate in the research. This strategy was chosen for its flexibility regarding sample size, allowing the inclusion of participants in varying numbers.

Although sequential sampling was used, finite population sampling methods were also applied, following parameters commonly adopted in opinion polls, epidemiological studies, and social research. The criteria established for sample size calculation included a 95% confidence level, a 5% margin of error, 80% statistical power, and an assumed variance of 50%, due to the lack of more specific data on the phenomenon under investigation. Additionally, a 10% increase was incorporated to compensate for potential sample losses, resulting in a minimum sample size of 385 individuals.

The sample size calculation was based on data from the Brazilian Institute of Geography and Statistics (IBGE) [34], which indicated that in 2022, there were 67.8 million people living in poverty and 12.7 million in extreme poverty in Brazil, totaling 80.5 million individuals in these conditions.

### 2.4. Healthcare System in Brazil

Brazil’s healthcare system operates under a dual model that encompasses both public and private sectors. The public system, known as the SUS, was established in 1988 by the Brazilian Constitution and guarantees universal, comprehensive, and free healthcare to all residents, including foreign nationals living in the country [35].

SUS is funded through taxes and contributions from all levels of government and provides access to services such as primary care, hospitalizations, specialized treatments, and medications. It is one of the largest publicly funded healthcare systems in the world, delivering services through a network of public hospitals and clinics, as well as private facilities contracted to serve SUS patients [36].

The private healthcare sector, in contrast, comprises health insurance plans and out-of-pocket payments for services. Private health insurance is often provided by employers as a benefit or purchased individually by those who can afford it. This sector offers access to a wider network of private hospitals and clinics, shorter waiting times for consultations and procedures, and more personalized care. Although only about 25% of the population has private health insurance, this segment accounts for a significant portion of healthcare expenditures in Brazil [37].

The primary difference between the public and private healthcare systems lies in the accessibility and quality of services. While the SUS is designed to provide equitable access to healthcare, resource constraints and high demand often lead to long waiting times and limited availability of specialized treatments [38].

By contrast, the private sector typically delivers faster and higher-quality services, but at a cost that renders it inaccessible to much of the population, particularly those in lower-income groups. This disparity underscores the persistent challenge of achieving health equity in Brazil, especially for socially vulnerable populations who depend heavily on SUS [35].

### 2.5. Survey Questionnaire and Data Collection Procedure

The study employed a questionnaire originally developed, validated, and applied in multiple studies by researchers from the National School of Public Health at Nova University of Lisbon (ENSP-UNL) [39,40,41,42]. For the Brazilian context, the questionnaire underwent a process of cultural adaptation and validation led by senior researchers using the Delphi technique [43]. This method involves collecting and refining expert opinions through iterative rounds of anonymous surveys, ensuring the instrument’s cultural relevance and appropriateness. Following this process, the questionnaire was named “Termômetro Social COVID-19: Opinião Social” (“COVID-19 Social Thermometer: Social Opinion”) [44].

The Delphi technique provided a robust framework for achieving expert consensus on key elements of the questionnaire, accounting for Brazil’s specific cultural and social nuances, and thereby enhancing the tool’s accuracy and applicability for assessing social perceptions during the pandemic. The instrument was hosted on the REDCap platform [45,46], managed by the Ribeirão Preto School of Nursing at the University of São Paulo (EERP-USP). Field interviewers, properly trained to minimize measurement bias, administered the questionnaire using mobile devices (smartphones and/or tablets). Each interview lasted approximately 20 to 30 min.

### 2.6. Data Collected and Study Variables

This study utilized sociodemographic and healthcare utilization variables to characterize the participants and assess inequalities in healthcare service utilization.

Sociodemographic Variables:

- Sex/Gender: Male, Female, Transgender, or Other;
- Age Group: 18–29 years, 30–59 years, or 60 years and older;
- Race/Ethnicity: White, Black/Brown, Indigenous, or Asian;
- Marital Status: Married/In a Stable Union, Widowed, Separated, or Single;
- Education Level: No Formal Education, Incomplete Primary Education, Complete Primary Education, Incomplete Higher Education, or Higher Education;
- Employment Status: Formal Employment, Informal Employment, Unemployed, Student, Retired, or Other;
- Monthly Income: No income, Less than one minimum wage, One to two minimum wages, Two to three minimum wages, or More than three minimum wages;
- Receipt of Government Assistance: Yes or No.

Healthcare Utilization Variables:

- Utilization of Public Healthcare Services: Yes or No;
- Utilization of Private Healthcare Services: Yes or No.

The age categorization (18–29, 30–59, and 60+ years) reflected lifecycle stages relevant to the analysis of social vulnerabilities during the pandemic. The 30–59 age group includes the consolidated economically active population, while the elderly (60+) are more vulnerable in terms of health. The broad age ranges were chosen to ensure statistical robustness [47,48,49]. These variables were essential for describing participant profiles and analyzing potential disparities in healthcare service utilization.

### 2.7. Statistical Analysis

The database was initially assessed for consistency and standardization using Microsoft Office Excel 2010. Subsequently, the data were organized into spreadsheets and imported into RStudio (version 4.3.1), where descriptive analyses were performed. Sociodemographic characteristics of the participants were described using absolute (n) and relative (%) frequencies.

To represent the geographic distribution of participants across Brazil’s five macroregions (North, Northeast, South, Southeast, and Midwest), thematic maps were created. Additionally, using ArcGIS software version 10.5, descriptive maps were produced to analyze the utilization of health services, focusing on the use of public and private services (yes/no responses) in each macroregion. Shapefile data provided by the Brazilian Institute of Geography and Statistics (IBGE) [50] were used to build the maps.

To analyze the influence of intersectionality on health service utilization (public and private), the Intersectionality Index—also known as the Jeopardy Index—was employed. This index combines multiple socioeconomic dimensions into a single score designed to reflect individuals’ positions within social systems and structures of power, privilege, and inequality [51]. This holistic approach allows for a broader understanding of social inequalities, recognizing that multiple factors can overlap and influence access to and use of health services [52].

The main exposure was constructed based on the principle of “multiple oppression” and classifies individuals into categories of greater or lesser social privilege. The index comprises three variables: sex/gender, race/color, and education level. Scores range from 0 to 5, with “0” assigned to the most privileged group (male, White, higher education) and “5” to the least privileged (cisgender women, transgender persons, Black or Brown individuals, and those with no formal education). These variables were selected based on strong evidence in the literature linking them to inequalities in health access and broader socioeconomic vulnerabilities [52,53,54,55,56,57].

To illustrate the distribution of participants according to the Jeopardy Index, a bar chart was created showing absolute (n) and relative (%) frequencies for each category (0 to 5). Subsequently, a proportion analysis was conducted for the outcomes “Use of Public Healthcare Services” and “Use of Private Healthcare Services,” stratified by the index. The results were graphically presented as line plots with error bars representing 95% confidence intervals (95% CI), allowing for assessment of the magnitude and statistical precision of the proportion estimates.

Finally, to examine the association between Jeopardy Index categories and health service utilization, binary logistic regression analyses were conducted for each outcome (public and private service use). Associations were expressed as odds ratios (ORs) with corresponding 95% confidence intervals (95% CIs). All analyses involving the Jeopardy Index were performed using Stata software (version 15.1).

### 2.8. Ethical Aspects

This study was approved by the Research Ethics Committee of the Ribeirão Preto School of Nursing of the University of São Paulo (EERP/USP), under Certificate of Submission for Ethical Appraisal (CAAE) no. 32210320.1.3001.5393. º32210320.1.3001.5393. The entire investigation was conducted in accordance with Resolution No. 466 of 12 December 2012 of the National Health Council, taking into account the relevant ethical and scientific foundations. Before starting the administration of the questionnaire, the Informed Consent Form (ICF) was read to the study participants, and only after their agreement and signature was the interview initiated. Informed consent was obtained from all individual participants included in the study.

## 3. Results

### 3.1. Demographics

Table 1 presents the sociodemographic data of the 3406 study participants, with a predominance of men at 60.0% (2045), followed by 38.2% (1302) women, 1.6% (53) transgender individuals, and 0.1% (5) of other genders. The most common age group was 30 to 59 years, accounting for 65.1% (2218), followed by 25.7% (877) aged 18 to 29, and 9.1% (311) aged 60 years or older. Regarding race/color, 72.2% (2460) identified as Black or Brown, followed by 22.0% (747) White, 3.0% (102) Asian, and 1.8% (63) Indigenous. In terms of marital status, 71.0% (2418) were widowed, separated, or single, while 29.0% (987) were married or in a stable union.

Education levels revealed that 43.5% (1483) had completed elementary school, 39.0% (1329) had completed high school, and 13.1% (447) had higher education or postgraduate degrees, while 4.3% (145) had no formal education. Regarding occupation, 39.0% (1328) were unemployed, 32.4% (1102) worked informally, and 14.3% (490) held formal jobs. Monthly income of less than one minimum wage was reported by 37.2% (1267), while 24.5% (833) had no income, and 21.9% (746) earned between one and two minimum wages. Additionally, 42.9% (1460) received some form of government assistance.

Regarding healthcare utilization, 91.4% (3112) reported using public healthcare services, 8.3% (284) did not, and 0.3% (10) did not respond. In contrast, only 6.5% (222) utilized private healthcare services, 92.9% (3167) did not, and 0.6% (17) did not respond.

These data indicate a predominantly adult population with low education and income levels, alongside significant dependence on social assistance programs, characterizing a high degree of socioeconomic vulnerability.

**Figure 1:**
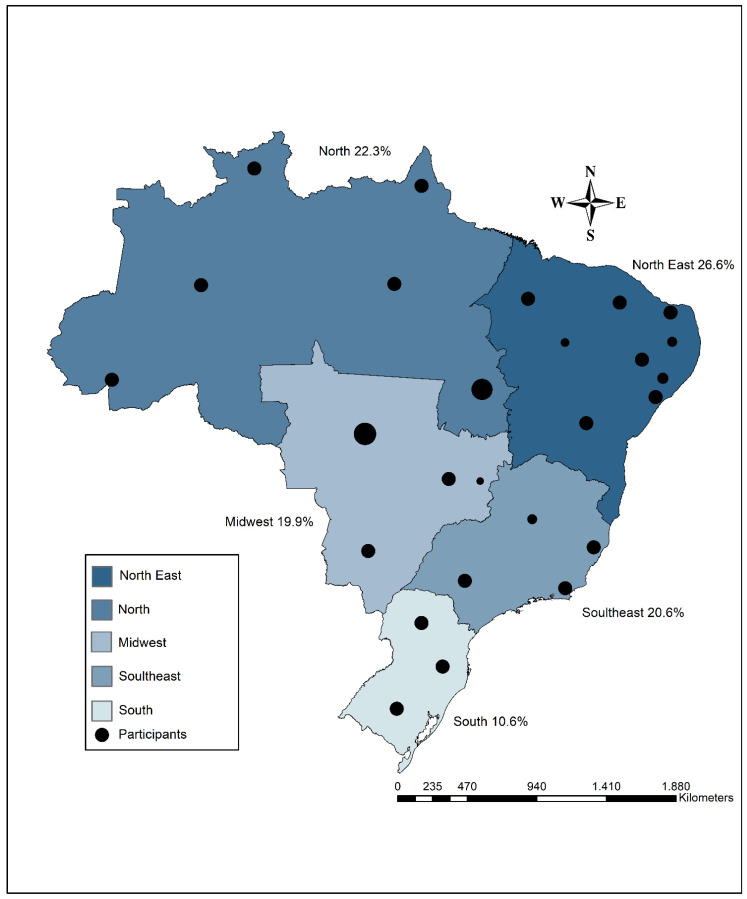
Geographic distribution of participants across the five major regions (macroregions) of Brazil.

Figure 1 shows the geographic distribution of participants across Brazil’s five major regions, revealing notable variations. The Northeast region has the highest number of participants, with 907 (26.6%). This is followed by the North region with 759 participants (22.3%), the Southeast with 703 participants (20.6%), the Midwest with 677 participants (19.9%), and the South with 360 participants (10.6%).

**Figure 2:**
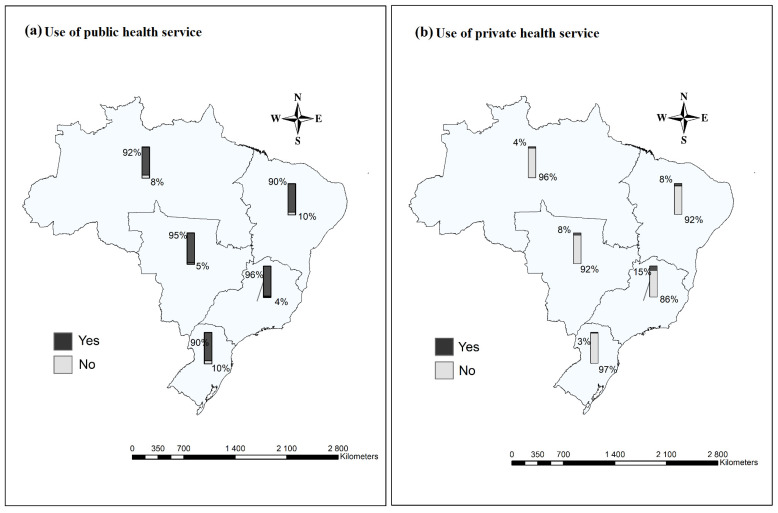
Prevalence of use of health services among vulnerable populations in the macroregions of Brazil during the COVID-19 pandemic, 2022–2023. Source: Prepared by the authors.

Across Brazil’s five macroregions, a striking majority of individuals rely on public health services, highlighting the central role of the Unified Health System (SUS) in providing care nationwide. As shown in Figure 2a, the proportion of people dependent on public health services remains consistently high: North (92.0%), Northeast (90.0%), Midwest (95.0%), Southeast (96.0%), and South (90.0%).

In contrast, access to private healthcare is limited across all regions, with notably low usage rates: North (4.0%), Midwest (8.0%), Southeast (15.0%), South (3.0%), and Northeast (8.0%), as illustrated in Figure 2b. These figures reflect the extensive reach and critical importance of Brazil’s public health infrastructure, especially in regions characterized by socioeconomic disparities and limited private sector presence, as emphasized in major scientific reviews and analyses of health access and equity in Brazil.

**Figure 3:**
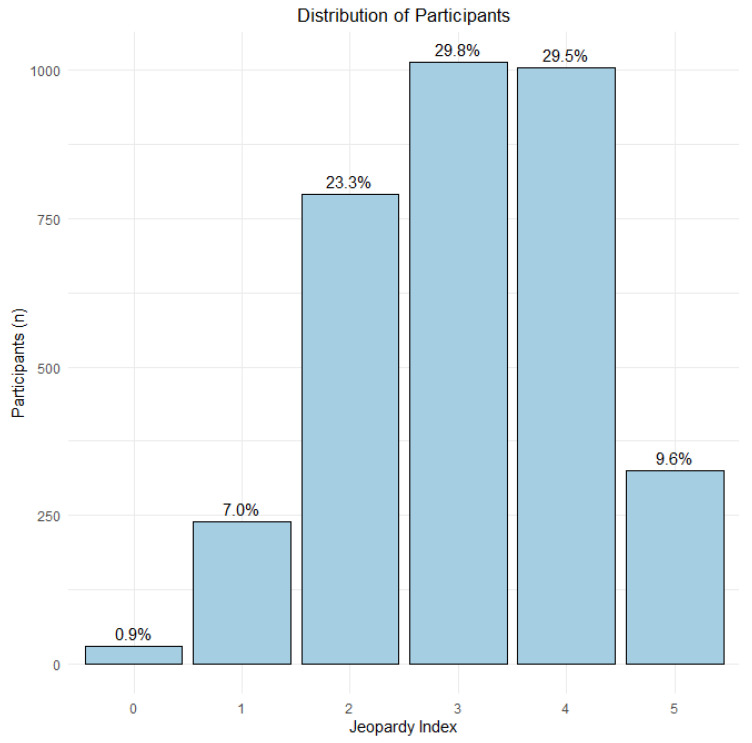
Distribution of participants across social vulnerability categories according to the Jeopardy Index. Legend: 0 (male, White, higher education); 1 (woman, White, higher education); 2 (woman, Asian or Black, incomplete higher education); 3 (cisgender woman/transgender person, Asian or Black, complete primary education); 4 (cisgender woman/transgender person, Black, incomplete primary education); 5 (cisgender woman/transgender person, Black, no formal education).

Figure 3 illustrates the stratification of participants according to the Jeopardy Index, highlighting the range of overlapping social vulnerabilities. The most privileged group—Profile 0 (male, White, higher education)—included only 29 individuals (0.9%). Profile 1 (female, White, higher education) comprised 239 participants (7.0%). Greater vulnerability was observed in Profile 2 (female, Black or Asian, incomplete higher education), with 791 individuals (23.3%). The highest levels of social disadvantage were found in Profile 3 (cisgender woman or transgender person, Black or Asian, completed elementary school; n = 1013, 29.8%), Profile 4 (cisgender woman or transgender person, Black, incomplete elementary school; n = 1004, 29.5%), and Profile 5 (cisgender woman or transgender person, Black, no formal education), representing the most marginalized group with 325 participants (9.6%). This gradient underscores profound disparities in vulnerability, emphasizing the intersection of gender, race, and educational attainment as key determinants of social privilege and disadvantage.

### 3.2. Regression and Proportions Results

The relationship between the Jeopardy Index categories and the use of SUS among socially vulnerable populations is presented in Table 2, revealing significant associations with race/color, gender, and education level. These findings indicate that less privileged groups (i.e., those with greater vulnerability) are more likely to rely on SUS.

The results show that, compared to the reference category (0), the most vulnerable group (5) had a significantly higher likelihood of using SUS (OR = 3.14; 95% CI = 1.34–7.35). This means that individuals in this category are 214% more likely to use public healthcare services compared to those in the reference group.

**Figure 4:**
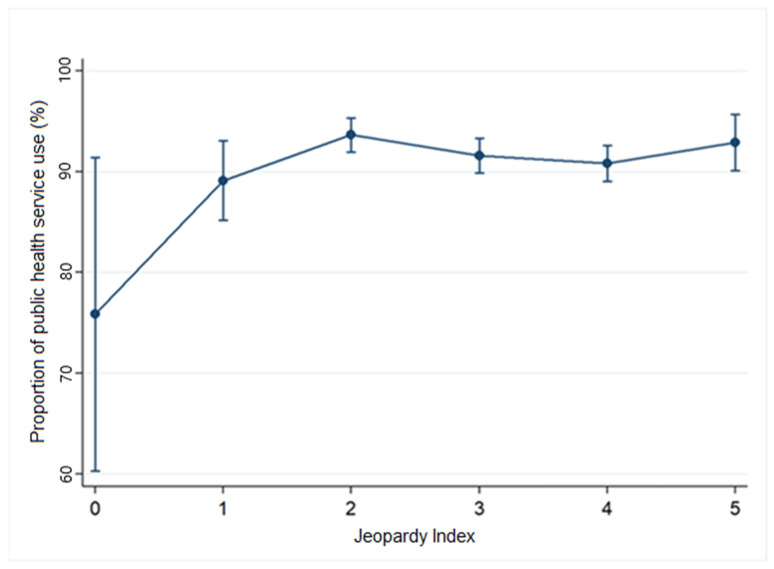
Proportion of individuals using public health services according to the Jeopardy Index, 2022–2023. Legend: Proportion of individuals who utilized public healthcare services (%) according to the Jeopardy Index. Categories range from 0 to 5, where: 0 = male, White, higher education; 1 = female, White, higher education; 2 = female, Black or Asian, incomplete higher education; 3 = cisgender woman or transgender person, Black or Asian, completed primary education; 4 = cisgender woman or transgender person, Black, incomplete primary education; 5 = cisgender woman or transgender person, Black, no formal education. Vertical bars represent 95% confidence intervals. Source: Prepared by the authors.

Figure 4 shows the proportion of participants who used public health services according to the Jeopardy Index, which ranges from 0 (highest social privilege) to 5 (highest intersectional vulnerability). There is a general increase in the use of public health services as social vulnerability rises, with about 75% usage at score 0 and stabilization above 90% starting from category 3.

The relationship between the Jeopardy Index categories and the use of SUS among socially vulnerable populations is presented in Table 3, where significant associations were observed.

The results indicated that, compared to the reference group (0), the most vulnerable group (5) was significantly less likely to use private health services (OR = 0.07; 95% CI = 0.02–0.20), which means that people in this group are 93% less likely to use private health services compared to the reference group.

**Figure 5:**
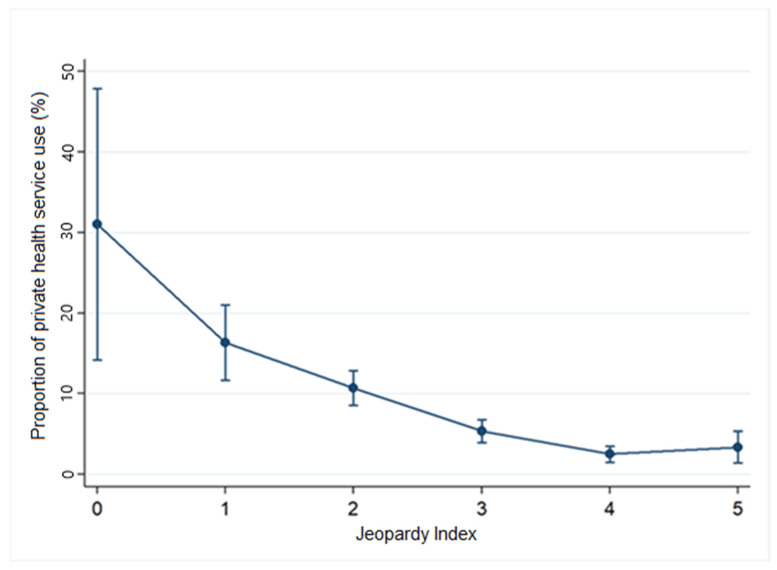
Proportion of individuals using private health services according to the Jeopardy Index, 2022–2023. Legend: Proportion of individuals who utilized private healthcare services (%) according to the Jeopardy Index. Categories range from 0 to 5, where: 0 = male, White, higher education; 1 = female, White, higher education; 2 = female, Black or Asian, incomplete higher education; 3 = cisgender woman or transgender person, Black or Asian, completed primary education; 4 = cisgender woman or transgender person, Black, incomplete primary education; 5 = cisgender woman or transgender person, Black, no formal education. Vertical bars represent 95% confidence intervals. Source: Prepared by the authors.

Figure 5 shows the proportion of participants reporting the use of private health services, stratified by the Jeopardy Index, which ranges from 0 to 5.

There is a clear downward trend in the use of private health services as the index categories increase. Approximately 54% of individuals in category 0 reported using private health services, while this proportion dropped to about 20% in category 5.

## 4. Discussion

This study aimed to investigate how intersectionality—considering sex/gender, race/color, and education level—influenced inequalities in the utilization of healthcare services by vulnerable populations during the COVID-19 pandemic in Brazil. Our findings show that individuals facing overlapping social disadvantages, such as women, transgender people, Black individuals, and those with lower educational attainment, experienced significant disparities in healthcare utilization. Without the SUS, which provides universal access, these populations would likely have been unable to access healthcare and would have been left behind. The private sector was predominantly used by men, white individuals, and people with higher levels of education.

The results revealed that the majority of socially vulnerable participants were men aged 30 to 59, Black or Brown, with only primary education, unemployed, and earning less than the minimum wage. Use of public healthcare services prevailed across all regions of Brazil, while use of private services was significantly lower—especially among women, transgender individuals, Black people, and those with no formal education. These groups were 214% more likely to use public healthcare and 93% less likely to use private healthcare. A direct relationship was observed between greater social vulnerability and dependence on the SUS, alongside limited access to private healthcare services.

The analysis of the sociodemographic data of the study participants revealed a profile of a socially vulnerable population, characterized by a predominantly male sample aged 30 to 59 years, Black or mixed-race, with elementary education, and unemployed. This profile reflects a well-documented pattern in the literature on social and racial inequalities in the country, intertwined with structural barriers that limit access to basic resources such as quality education, formal employment, and stable income [58,59,60].

According to IBGE [61,62], Black and mixed-race individuals constitute the majority of the population living in extreme poverty, with limited access to education and employment opportunities. This reality is further compounded by labor market discrimination, which leads to economic exclusion and dependence on social programs and essential public services such as healthcare [63,64].

An analysis of regional data shows that people across all parts of the country—especially in the Midwest and Southeast—heavily depend on the SUS. This is likely due to greater SUS coverage in these regions. According to Chaves et al. [65], 62.5% of the Midwest’s macroregions have high coverage in primary care, medium coverage in hospital care, low quality in both, and high resolution capacity of the SUS. In the Southeast, 81.25% of macroregions are characterized by high primary care coverage, medium hospital care coverage, and high quality.

Conversely, the lower use of private health services in Brazil’s macroregions also reflects inequalities in infrastructure and available resources. While the SUS predominates in healthcare provision across all regions, the North and Northeast face additional challenges due to the historical lack of investment in healthcare infrastructure [66]. Regional inequality remains a significant barrier, with the North and Northeast experiencing greater difficulties due to geographic distance and the insufficient number of Basic Health Units (UBSs), particularly in rural areas. These regions require greater investment to improve the organizational accessibility of healthcare services [67,68].

Vulnerable populations—such as informal workers, Black individuals, and residents of peripheral areas—faced significant barriers to accessing hospital services, diagnostic tests, and Intensive Care Unit (ICU) beds. The pandemic exposed the fragility of health policies and highlighted the urgent need for structural investments in the SUS, particularly in regions with inadequate infrastructure [69].

As a universal healthcare model, the SUS represents the main strategy to reduce inequalities in access to healthcare in Brazil. Established by the 1988 Constitution, it guarantees free and comprehensive healthcare to the population, playing a crucial role in serving vulnerable groups who would otherwise be excluded from the system [70]. The reach of the SUS is especially critical in regions where private services are scarce, acting as the only viable option for thousands of Brazilians [71].

However, reliance on the SUS underscores the structural limitations of social inclusion in the country. Populations in socially vulnerable situations—such as low-income individuals, the unemployed, and residents of peripheral areas—often lack the resources to afford private services, reinforcing their dependence on the public system [72]. Thus, economic and social inequality are directly reflected in the demand for the SUS, emphasizing the need for continuous investment to expand its capacity and ensure quality care for all [73,74].

The data show that women, trans individuals, Black people, and those with low levels of education were 164% more likely to use public health services during the COVID-19 pandemic and 93% less likely to use private health services. This disparity is not limited to financial barriers alone but also includes institutional and social obstacles that perpetuate the exclusion of these populations from healthcare access [75]. Factors such as limited resources, lower education, and residence in peripheral areas significantly contribute to the reliance on public health services, which, although essential, face limitations in infrastructure and human resources [76].

Moreover, issues of discrimination and prejudice exacerbate this scenario. Black women, for example, often report differential treatment and discriminatory attitudes in healthcare settings, leading to greater dissatisfaction and a lower perceived quality of services [77]. For trans individuals, the challenges include neglect, lack of preparedness among healthcare professionals, and institutionalized transphobia—factors that create negative experiences and discourage the use of services, especially private ones [78]. These inequalities underscore the need for public policies that address discrimination and expand equitable access to quality healthcare for historically marginalized populations.

The association between race and reduced access to private healthcare services highlights the persistent impacts of structural racism on health in Brazil. Black and Brown populations face historical disadvantages that result in lower educational attainment, higher unemployment rates, precarious working conditions, and greater exposure to environmental and occupational risks [79].

Racial inequalities create barriers to healthcare access, reducing opportunities for prevention and treatment, and contributing to poorer living conditions and a higher prevalence of preventable diseases. Black individuals, in particular, experience higher rates of untreated chronic conditions, such as hypertension and diabetes, reflecting the impact of structural racism on unequal access to healthcare [80].

It is important to highlight that the pandemic also exposed intersecting inequalities related to gender and gender identity. Studies [81,82] show that women—particularly Black women—were more exposed to the risk of infection due to their overrepresentation in informal labor and caregiving sectors, with limited access to personal protective equipment (PPE) and adequate medical care. These factors intensified the exclusion of already historically marginalized groups.

The direct relationship observed between greater social vulnerability and dependence on public health services—along with limited use of private services—confirms the role of the public system in mitigating inequalities and underscores the need for a more equitable approach to health planning in Brazil. Therefore, it is essential to strengthen the SUS as a central strategy for promoting health equity. Studies [83,84] suggest that public policies involving increased funding, decentralization, and the strengthening of primary care can help mitigate the impacts of the inequalities evidenced in this study.

One limitation of this study is the representativeness of the sample, which may not capture all the social and geographical variables that influence access to healthcare in Brazil. The research focused on a specific population, predominantly men, Black or mixed-race individuals with low education and income, which may exclude other vulnerable groups, such as people with disabilities or the elderly. Additionally, the analysis of regional inequalities in healthcare access did not sufficiently address remote areas or those with distinct sociodemographic characteristics. The lack of updated data and the inability to obtain information from all regions of the country also limit the scope of the study.

Moreover, there are possible analytical limitations related to the data and methods used, including the cross-sectional nature of the analysis, which restricts the ability to infer causality and capture temporal changes. Residual biases may also remain despite efforts to control for confounders, and there are inherent limitations in the construction and application of the Jeopardy Index as a measure of intersectional social vulnerability.

This study is subject to methodological limitations that warrant consideration. First, the retrospective nature of data collection—spanning from May 2022 to October 2023, after the most acute phases of the pandemic—introduces the potential for recall bias. Substantial evidence from recent research indicates that memory of pandemic-related events is often distorted, with subjective interpretations and selective recall affecting the accuracy of retrospective reports, even when validated and culturally adapted instruments are used.

Second, the categorization of participants into three broad age groups (18–29, 30–59, and 60 years or older), while necessary to maintain statistical power, may obscure important heterogeneity within these categories—particularly in the wide 30–59 age range. This limitation aligns with findings in the literature, which advocate for more granular subgroup analyses in future studies with larger sample sizes to better elucidate age-related differences in healthcare access and outcomes.

Despite these constraints, the study’s findings provide a consistent and reliable overview of prevailing trends, supported by rigorous methodology and validated measurement tools. It is also important to note that, as an association study employing the Jeopardy Index for intersectional analysis, the results should be interpreted as predictive rather than causal, in accordance with best practices for observational research as outlined in major scientific publications. Future research should aim to address these limitations by incorporating larger, more diverse samples and longitudinal designs to further advance the understanding of healthcare inequalities in the post-pandemic context.

This study advances knowledge by revealing the complex relationship between social inequalities and the utilization of healthcare services in Brazil, highlighting how factors such as race, gender, class, and education directly affect equity in access to care. Integrating public health strategies with broader social initiatives—such as poverty reduction, education, and efforts to combat structural racism—can create positive synergies for promoting social justice. Initiatives that address the social determinants of health are essential to tackling the root causes of inequality and fostering a more equitable society [85].

The COVID-19 pandemic acted as a catalyst, exposing and intensifying pre-existing social and structural inequalities in Brazil. While the SUS played a central role in responding to the pandemic and mitigating inequalities, it was severely strained by high demand and limited resources [73,74]. In this context, investing in the strengthening of the SUS, integrating intersectoral policies, and addressing social and institutional barriers are critical to promoting health equity in the post-pandemic era and preparing for future health crises.

## 5. Conclusions

This study highlights the profound intersectionality of social inequalities in Brazil and their impact on healthcare utilization during the COVID-19 pandemic. Our findings confirm that individuals facing multiple social disadvantages—such as women, Black individuals, transgender people, and those with lower levels of education—were disproportionately dependent on the SUS. The pandemic amplified existing disparities, further exposing barriers to healthcare access for vulnerable populations, particularly in regions with insufficient infrastructure. These findings underscore the critical role of the SUS in mitigating inequalities, as it provided essential care to populations who would otherwise have been excluded. However, the overreliance on the SUS also points to the urgent need for sustained investment to strengthen its capacity—especially in underserved areas—and to address broader structural determinants of health, such as poverty, education, and racial discrimination.

The study also highlights how gender inequality and social exclusion limit access to healthcare, with marginalized groups facing greater barriers to private services. Addressing these challenges requires not only the strengthening of public health services but also the implementation of policies that tackle the social determinants of health and promote equity in healthcare utilization. The pandemic served as a catalyst, bringing these longstanding issues to light. It is evident that intersectoral efforts are essential to building a more inclusive and equitable healthcare system in Brazil. Strengthening the SUS, reducing regional disparities, and confronting the root causes of inequality will be crucial to ensuring that all populations—regardless of their social background—can access quality healthcare in future health crises.

## Figures and Tables

**Table 1:** Sociodemographic characteristics of study participants, Brazil, 2022–2023 (n = 3406).

Variables	n (%)
**Sex/Gender**	
Male	2045 (60.0)
Female Transgender	1302 (38.2) 53 (1.6)
Others	5 (0.1)
No reply	1 (0.0)
**Age (years)**	
18 to 29	877 (25.7)
30 to 59	2218 (65.1)
60 years or older	311 (9.1)
**Race/color**	
White	747 (22.0)
Black/brown	2460 (72.2)
Indigenous	63 (1.8)
Asian	102 (3.0)
No reply	34 (1.0)
**Marital status**	
Married or in a stable union	987 (29.0)
Widowed, separated, or single	2418 (71.0)
No reply	1 (0.0)
**Education**	
No formal education	145 (4.3)
Complete primary education	1483 (43.5)
Completed high school	1329 (39.0)
Complete university or post-graduate degree	447 (13.1)
No reply	2 (0.1)
**Occupation/Employment**	
Formal employment	490 (14.3)
Informal employment	1102 (32.4)
Unemployed	1328 (39.0)
Student	143 (4.2)
Retired/Pensioner	187 (5.5)
Others	155 (4.6)
No reply	1 (0.0)
**Monthly income**	
No income	833 (24.5)
Less than 1 minimum wage	1267 (37.2)
From 1 to 2 minimum wages	746 (21.9)
From 2 to 3 minimum wages	184 (5.4)
Above 3 minimum wages	116 (3.4)
No reply	260 (7.6)
**Receives government aid**	
Yes	1460 (42.9)
No	1943 (57.0)
No reply	3 (0.1)
**Utilization of Public Healthcare Services**	
Yes	3112 (91.4)
No	284 (8.3)
No reply	10 (0.3)
**Utilization of Private Healthcare Services**	
Yes	222 (6.5)
No	3167 (92.9)
No reply	17 (0.6)

**Table 2:** Results of the binary logistic regression analysis examining the association between public healthcare service utilization and the Jeopardy Index categories.

Variable	Odds Ratio [95% CI]	Marginal Effect	*p*-Value
0—Male, White, Higher Education	-	0.759	Ref
1—Woman, White, Higher Education	4.69 [1.91–11.5]	0.891	0.001 **
2—Woman, Asian or Black, Incomplete Higher Education	3.45 [1.43–8.33]	0.936	0.006 **
3—Cisgender Woman/Transgender person, Asian or Black, Complete Primary Education	3.15 [1.31–7.58]	0.916	0.010 *
4—Cisgender Woman/Transgender person, Black, Incomplete Primary Education	4.16 [1.60–10.7]	0.908	0.003 **
5—Cisgender Woman/Transgender person, Black, No Formal Education	3.14 [1.34–7.35]	0.929	0.008 **

Legend: OR: odds ratio; 95% CI: 95% confidence interval; *p* < 0.05 * and *p*-value < 0.01 **.

**Table 3:** Results of the binary logistic regression analysis examining the association between private healthcare service utilization and the Jeopardy Index categories.

Variable	Odds Ratio [95% CI]	Marginal Effect	*p*-Value
0—Male, White, Higher Education	-	0.479	Ref
1—Woman, White, Higher Education	0.26 [0.11–0.06]	0.210	0.002 **
2—Woman, Asian or Black, Incomplete Higher Education	0.12 [0.05–0.28]	0.128	0.00 ***
3—Cisgender Woman/Transgender person, Asian or Black, Complete Primary Education	0.05 [0.02–0.13]	0.067	0.00 ***
4—Cisgender Woman/Transgender person, Black, Incomplete Primary Education	0.04 [0.20–0.98]	0.035	0.040 *
5—Cisgender Woman/Transgender person, Black, No Formal Education	0.07 [0.02–0.20]	0.053	0.00 ***

Legend: OR: odds ratio; 95% CI: 95% confidence interval; *p* < 0.05 *, *p*-value < 0.01 **, and *p*-value < 0.001 ***.

## Data Availability

The datasets used and/or analyzed during the current study are available from the corresponding author on reasonable request.
